# ACL reconstruction with femoral and tibial adjustable versus fixed-loop suspensory fixation: a retrospective cohort study

**DOI:** 10.1186/s13018-022-03128-y

**Published:** 2022-04-19

**Authors:** Sebastian Schützenberger, F. Keller, S. Grabner, D. Kontic, D. Schallmayer, M. Komjati, C. Fialka

**Affiliations:** 1 Department of Orthopaedic Surgery and Traumatology, AUVA Traumacenter Meidling, Kundratstrasse 37, 1120 Vienna, Austria; 2 Department of Orthopaedic Surgery, Sacred Heart of Jesus Hospital, Vienna, Austria; 3 Department for Traumatology, Sigmund Freud Medical University, Vienna, Austria

**Keywords:** ACL reconstruction, Adjustable-loop, Fixed-loop, Suspensory fixation, All-inside

## Abstract

**Background:**

Cortical suspensory fixation (CSF) devices gain more and more popularity as a reliable alternative to interference screws for graft fixation in anterior cruciate ligament (ACL) reconstruction. Adjustable-loop fixation may be associated with increased anterior laxity and inferior clinical outcome. The purpose of the study was to compare anterior laxity and clinical outcome after minimally invasive all-inside ACL reconstruction using an adjustable-loop (AL) to a standard technique with a fixed-loop (FL) CSF device.

**Methods:**

Patients who underwent primary single-bundle ACL reconstruction with a quadrupled hamstring autograft at a single institution between 2012 and 2016 were reviewed. In the AL group minimally invasive popliteal tendon harvesting was performed with an all-inside approach (femoral and tibial sockets). In the FL group a traditional anteromedial approach was used for tendon harvesting and a femoral socket and full tibial tunnel were drilled. An objective clinical assessment was performed with Telos x-rays and the International Knee Documentation Committee (IKDC) Objective Score. Patient-reported outcomes (PRO) included the IKDC Subjective Score, the Lysholm Knee Score, the Knee Injury and Osteoarthritis Score (KOOS) and the Tegner Activity Scale.

**Results:**

A total of 67 patients were enrolled in this retrospective study with a mean follow-up of 4 (± 1.5) years. The groups were homogenous at baseline regarding age, gender, and the time to surgery. At follow-up, no statistically significant differences were found regarding anterior laxity (AL: 2.3 ± 3 mm vs. FL: 2.3 ± 2.6 mm, *p* = 0.981). PRO scores were comparable between the AL and FL groups (IKDC score, 84.8 vs. 88.8, *p* = 0.185; Lysholm 87.3 vs. 89.9, *p* = 0.380; KOOS 90.7 vs. 91.4, *p* = 0.720; Tegner 5.5 vs. 6.2, *p* = 0.085). The rate of saphenous nerve lesions was significantly lower in the AL group with popliteal harvesting of the tendon (8.3% vs. 35.5%, *p* = 0.014).

**Conclusion:**

The use of an adjustable-loop device on the femoral and tibial side led to similar stability and clinical results compared to a fixed-loop device.

## Background

Anterior cruciate ligament (ACL) reconstruction using all soft tissue grafts relies on secure graft fixation to facilitate a rehabilitation program aiming for early restoration of knee motion and strength. In recent years, suspensory fixation has gained more and more popularity as an alternative to the use of interference screws, because it may improve tendon-to-bone healing (1,2) and it avoids damage to the graft during screw insertion (3,4). Furthermore, the insertion of interference screws leads to a significant initial enlargement of the bone tunnel at the time of the surgery and results in larger tibial tunnels after 2 years compared to extracortical fixation (5,6). Another technical issue that might contribute to a loss of pullout strength, when using interference screws for graft fixation, is screw divergence (7,8).

The first generation of suspensory devices consisted of a fixed-loop (FL) attaching the graft to a metallic button. Even though FL fixation provides excellent stability with high fixation strength (9,10), it has drawbacks too. Fixed-loop devices require accurate measurement during tunnel preparation because of their predetermined loop length. The femoral socket is drilled 6-10 mm longer to enable the “flip” movement of the button, resulting in a cavity above the graft after it is tensioned. This technique may contribute to the so-called bungee and windshield wiper effects resulting in a higher risk of tunnel widening (11). The second generation are adjustable-loop (AL) suspensory devices with an adjustable 1-way locking mechanism relying on friction between sutures to maintain a certain length. AL fixation facilitates the adjustment of graft tension and re-tensioning after passive cycling of the knee. Furthermore, better filling of the bone tunnels is possible, reducing the dead space (9,11,12).

However, adjustable-loop devices may lengthen under cyclic loading as reported in biomechanical studies(13–18), causing laxity of the graft. This issue may be reduced with re-tensioning and knot tying of the AL construct (9,13,15). The all-inside (AI) ACL reconstruction technique has been demonstrated to result in less post-operative pain and is bone conserving (19,20), but relies on AL suspensory fixation on the femoral and tibial side. Good biomechanical and clinical results are reported for AI ACL reconstruction in various studies (21). Nevertheless, Bressy et al. raised awareness for insufficient stability with the AI technique (22).

The aim of this study was to compare anterior laxity and clinical outcomes between AL and FL suspensory fixation on the femoral and tibial side using a 4-stranded hamstring autograft. The hypothesis was that the minimally invasive all-inside ACL reconstruction technique with AL fixation and a tibial socket would be comparable to a technique with FL fixation and a full tibial tunnel in terms of stability and clinical outcome.

## Methods

### Population

For this cohort study, we performed a database search for all surgically treated patients with ACL tears with clinical instability. A total of 396 patients were operated between 2012 and 2016, of which 351 patients fulfilled the inclusion criteria (primary ACL reconstruction with quadrupled semitendinosus tendon with suspensory fixation on the femoral and tibial side). There has already been a publication out of this patient collective, covering a different research question with different subsamples (23). All patients with bilateral ACL reconstruction (n = 12), complex meniscal lesions (subtotal/total meniscectomy, complete meniscal root avulsions and complete radial tears—n = 15), as well as multiligamentous injuries (n = 10) and high-grade chondral lesions requiring surgical treatment (n = 9) were excluded from the study. A total of 305 patients were invited to participate in the study, 75 patients gave their informed consent and were retrospectively included to the study. Patients who underwent revision ACL surgery or were newly diagnosed with a re-rupture of the graft (n = 8) were also excluded, leaving 67 eligible patients.

The acquisition of the data for the follow-up examination was conducted prospectively and included a standardized clinical assessment with measurement of the active range of motion (ROM) of both knees with goniometry, the thigh circumference, as well as all the other parameters of the International Knee Documentation Committee (IKDC) Objective Score. The patients were asked if they suffer from residual hyposensitivity in the innervation area of the infrapatellar branch of the saphenous nerve. Patient-reported outcomes at the time of follow-up were evaluated prospectively using written questionnaires, including the IKDC Subjective Score, the Lysholm Knee Score, the Knee Injury and Osteoarthritis Outcome Score (KOOS) and the current Tegner Activity Scale. In addition, the medical records of all patients were assessed to determine demographics and details of the surgical procedure. Subsequently, knee laxity was evaluated with Telos stress x-rays and each patient had a magnetic resonance imaging (MRI) scan to detect a potential rerupture of the ACL graft.

### Surgical technique

**Fig. 1:**
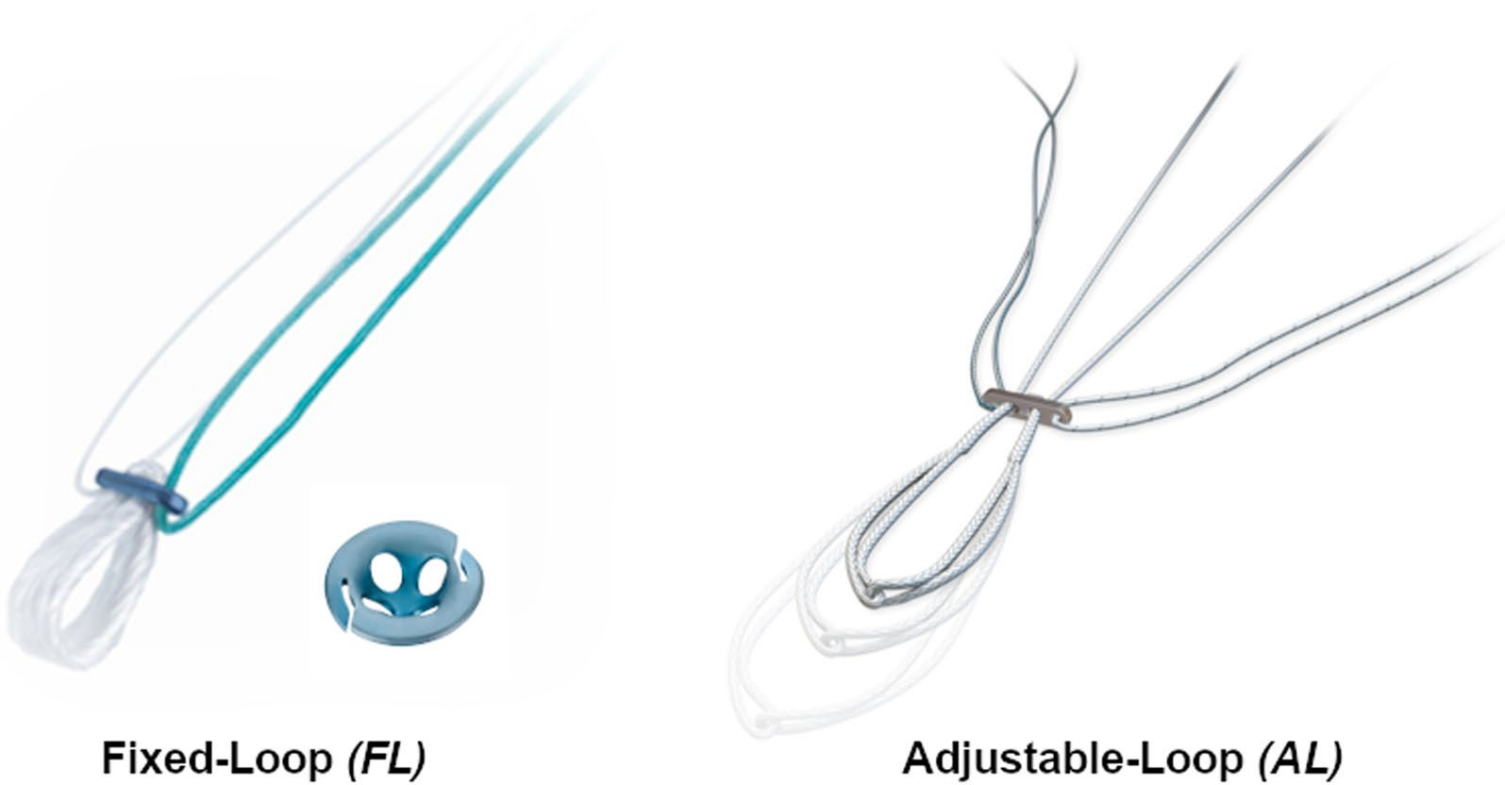
Cortical Suspensory Fixation Devices – In the *FL group* a fixed loop (Suture Plate, B.Braun-Aesculap, Tuttlingen, Germany) was used on the femoral side and on the tibial side (Suture Disk, B.Braun-Aesculap, Tuttlingen, Germany). In the *AL group* two adjustable loops were used on the femoral and the tibial side (ACL TightRope RT, Arthrex, Naples, FL, USA). *(images used with permission of B.Braun-Aesculap and Arthrex)*

All operations were performed by one out of six surgeons specialized in knee surgery at our hospital, either with a fixed-loop (FL) system (Position ACL, B.Braun-Aesculap, Tuttlingen, Germany) or with an adjustable-loop (AL) technique (All-Inside ACL, Arthrex, Naples, FL, USA) according to the recommendations of the manufacturer (Fig. 1). Prior to the actual ACL reconstruction, meniscus and cartilage pathologies were treated arthroscopically, if necessary. A combination of anatomical landmarks (namely the remnants of the ACL, the lateral intercondylar ridge, the apex of the deep cartilage and the anterior horn of the lateral meniscus) were used to guide tunnel placement during surgery.

### Fixed-loop fixation

The semitendinosus (ST) tendon alone was harvested through an incision over the pes anserinus superficialis. The two free ends were whip-stitched using non-resorbable high-strength suture material (Orthocord #2, DePuy Synthes Mitek Sports Medicine, Raynham, MA, USA), then the tendon was folded into an M-formation and inserted into the closed loop (Suture Plate, B.Braun-Aesculap, Tuttlingen, Germany) to obtain a four-stranded graft. The femoral socket was drilled according to anatomical landmarks through the anteromedial portal at 110°–120° of flexion. The tibial tunnel was created in the middle of the remaining distal stump of the ACL. The graft was pulled transtibially into the femoral socket. Tibial fixation was performed using a suture button (Suture Disk, B.Braun-Aesculap, Tuttlingen, Germany). After passive cycling of the knee joint the transplant was tensioned in 30° of knee flexion by twisting of the tibial button.

### Adjustable-loop fixation

**Fig. 2:**
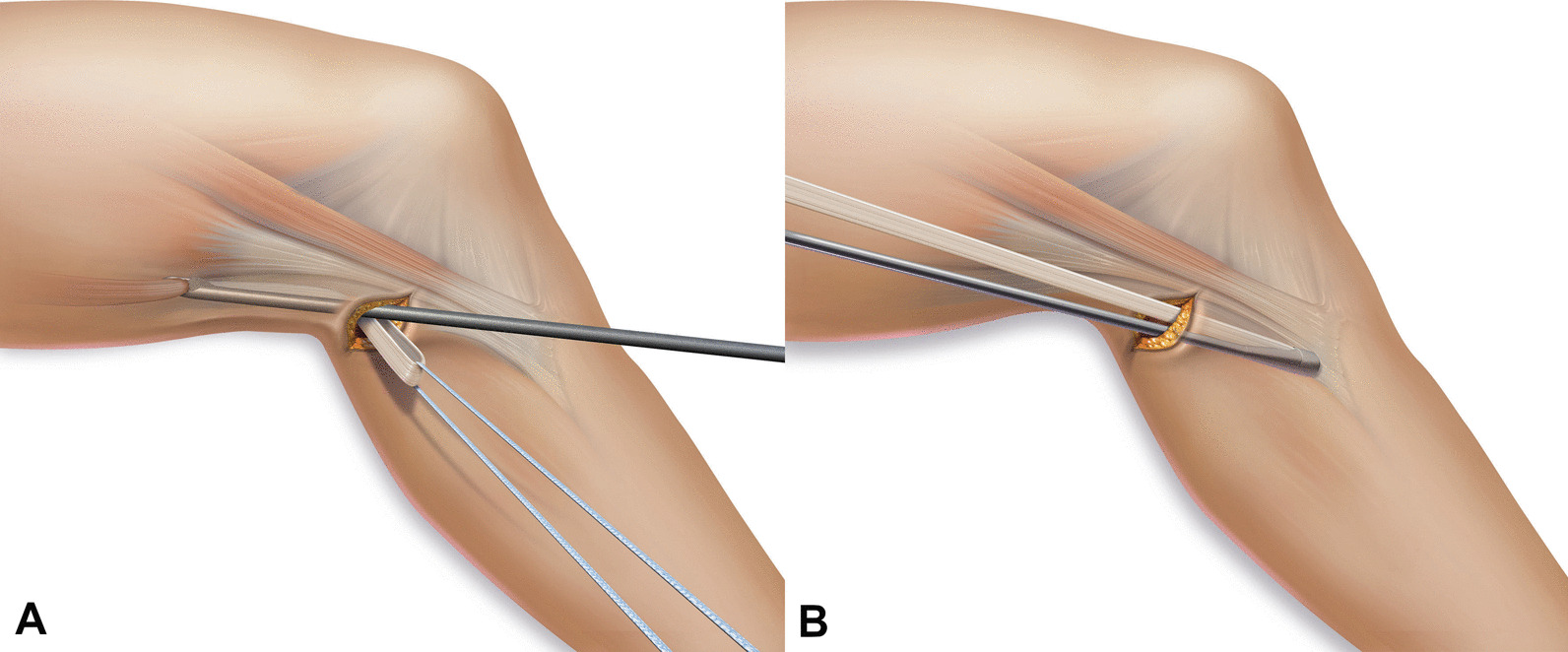
Minimally Invasive Popliteal Hamstring Harvest—After creating a 15–20 mm transverse incision centred on the ST tendon in the flexion fold the tendon was located and placed on a traction suture. **A** Retrograde harvesting of the tendon with an open stripper. **B** Anterograde harvesting of the tendon with a short closed stripper. *(Images used with permission of Arthrex)*

The ST tendon was harvested through a minimally invasive popliteal approach (24). (Fig. 2) The tendon was symmetrically folded over two adjustable-length loops (ACL TightRope RT, Arthrex, Naples, FL, USA) in order to obtain a four-stranded graft. The graft was secured with two sutures at the tibial end and two sutures at the femoral end of the graft (FiberWire # 2; Arthrex, Naples, FL, USA) (25). A femoral and tibial socket was created at the anatomic ACL insertion site with an outside-in technique using a retrograde drill (FlipCutter, Arthrex, Naples, FL, USA). The graft was inserted through the AM portal and shuttled into the bone sockets via pull-through sutures. Cortical buttons were flipped, and the graft tensioned with the knee in extension. The knee was then passively cycled and the graft re-tensioned as required. Finally, the tensioning strands of both adjustable-loops (on the femoral and tibial side) were knotted with an arthroscopic knot pusher to reduce the risk of loop slippage (13).

### Rehabilitation protocol

Post-operatively, no brace was used in the group of patients with isolated ACL tears (n = 34) and full weight bearing was allowed after 2–4 weeks (after quadriceps control had been regained). The patients with meniscal repair (n = 33) were restricted to partial weight-bearing for 4 weeks and flexion was limited to 60° for 4 weeks and to 90° for another 2 weeks with a knee brace.

Physiotherapy was started on the first post-operative day while still on admission. Return to sports was recommended not earlier than 9–12 months post-operatively, depending on thigh circumference, rehabilitation milestones and confidence to return to sports.

The same standardized rehabilitation protocol was used for both treatment groups.

### Radiological measurements

**Fig. 3:**
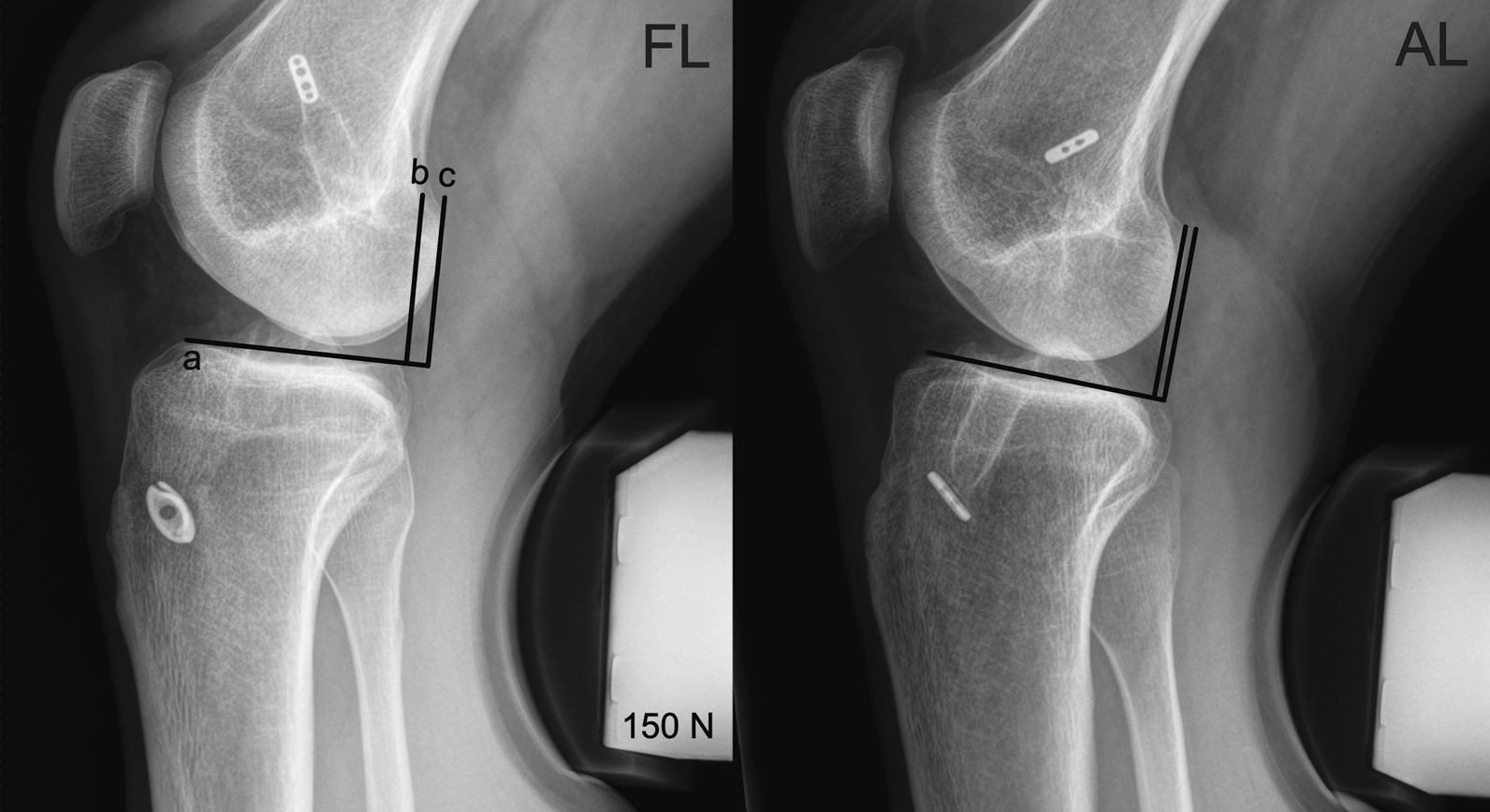
Anterior laxity measurement – Sagittal stress x-ray with the Telos device. **(FL)** 31-year-old male patient after FL ACL reconstruction. **a** Tangent to the medial tibial plateau, **b** line through the posterior edge of the femoral condyles and perpendicular to the tangent, **c** line through the posterior edge of the medial tibial plateau and perpendicular to the tangent. **(AL)** 26-year-old male patient after AL ACL reconstruction

Dynamic passive Lachman X-rays of both knees (operated knee and contralateral healthy knee) were taken by the department’s radiology technicians on a Telos GA-III/E device (Telos GmbH Laubscher, Holstein, Switzerland) at 150 N. Differential (Dif AD) measurements (operated knee versus healthy contralateral knee) for the anterior drawer of the medial compartment were estimated, as described before (26) (Fig. 3).

All participants underwent MRI (1,5 T, Avanto, Siemens AG Healthcare, Erlangen, Germany) to evaluate the integrity of the anterior cruciate ligament graft with a 15-channel phased-array transmit/receive knee coil (Siemens AG Healthcare, Erlangen, Germany) with the following sequences: sagittal (sag.) T1-weighted Turbo Spin Echo (TSE); sag., coronal (cor.), axial (ax.) T2-weighted Blade with Fat Saturation (FS); sag. Proton Density (PD) with Spatial Phase Coding (SPC).

### Statistical analysis

Sample size analysis resulted in a need to recruit 24 patients per group to detect a clinically significant group difference (2.5 mm, SD 3.0 mm) in side-to-side difference of the anterior translation, given a significance level of 0.05 and a power of 0.80 (27). Statistical analyses were done using the free software environment R version 3.6.3 on a PC running Linux Ubuntu version 16.04.6 LTS (28). Descriptive statistics are given as counts, percentages, or means, and standard deviations (SD) or ranges as appropriate. For inferential statistics, t-test and χ2-test were used, and p-values for tests performed are given rounded to three decimal places.

## Results

### Population

This study included 67 patients (48 male and 19 female) with a mean age of 26.9 ± 8.7 years (range 13–50). The mean follow-up was 45.1 ± 18.3 months (range 19–85). The groups were homogenous at baseline regarding age, gender, and the time to surgery. As the fixed-loop technique was gradually replaced by the adjustable-loop technique the time to follow-up was significantly shorter in the AL group. Forty-three patients (64%) had a meniscal injury at the time of the primary surgery, which was treated with meniscus repair in 33 cases and with partial meniscectomy in 10 cases. One patient had an early post-operative infection and was successfully treated with arthroscopic lavage. A further 11 patients underwent subsequent surgery: 7 for a partial meniscectomy, 3 for resection of a cyclops lesion and 1 for tibial suture button removal. The complication rate was higher in the FL group (12.9% vs. 5.6%).

At the time of follow-up, 8 ACL reconstructions (9.2%) had failed: 2 patients (5.2%) in the AL and 6 patients (16.2%) in the FL group (*p* = 0.153) (Table 1).

**Table 1:** Baseline characteristics

	Adjustable loop (n = 36)		Fixed loop (n = 31)	*P* value
Age (years)	27.5 ± 9.5	26.3 ± 7.8		0.578
Male, n (%)	24 (67%)	24 (77%)		0.419
Time to surgery (days)	116 ± 56	84 ± 78		0.067
Time to follow-up (months)	32 ± 7	61 ± 14		< 0.001
Meniscal tears, n (%)	24 (67%)	19 (61%)		0.840
Complications, n (%)	2 (5.6%)	4 (12.9%)		0.506
Thrombosis	0	1		
Infection	1	0		
Cyclops lesion	1	2		
Suture button removal	0	1		

### Radiographic laxity measurements

**Fig. 4:**
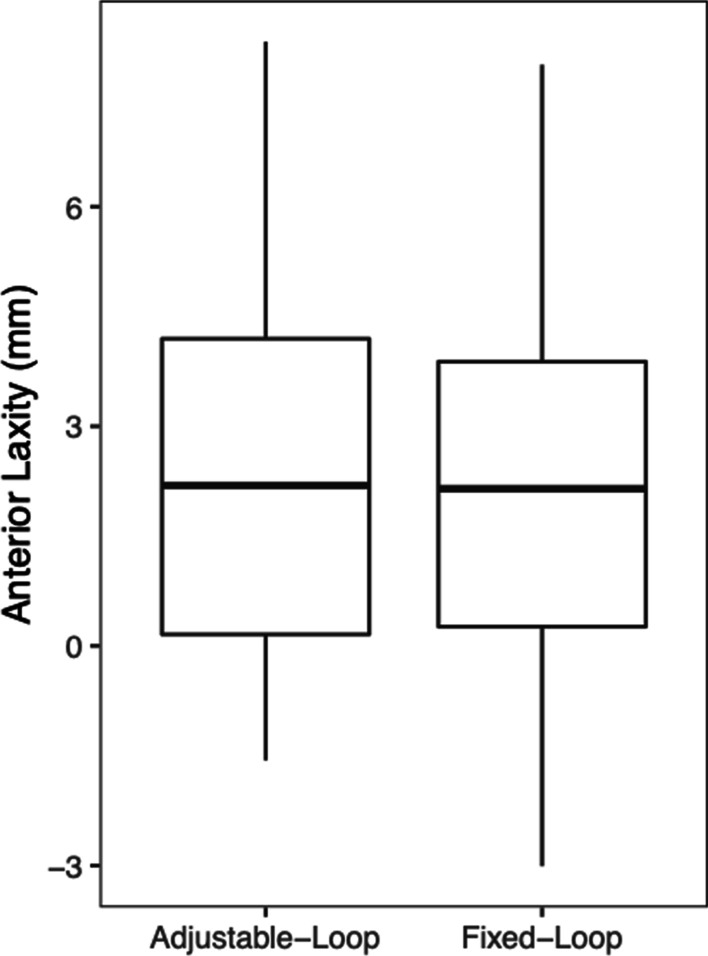
Anterior laxity measured by Telos stress X-rays

The mean differential anterior drawer (Dif AD) was 2.3 ± 3 mm in the FL group and 2.3 ± 2.6 mm in the AL group. There was no statistically significant difference regarding the anterior translation between the groups (*p* = 0.981). (Fig. 4).

### Clinical results

#### Clinical assessment

No significant differences were observed between the groups in terms of side-to-side difference in ROM, thigh circumference and objective IKDC (Table 2).

**Table 2:** Objective clinical assessment

	Adjustable loop (n = 36)	Fixed loop (n = 31)	*P* value
Range of Motion (°)			
Extension deficit	− 1.25 ± 2.77	− 0.65 ± 2.14	0.318
Flexion deficit	− 2.36 ± 3.68	− 2.42 ± 3.85	0.950
Thigh Circumference (cm)	− 1.57 ± 1.63	− 0.98 ± 1.37	0.117
ROM and thigh circumference measurements are displayed as difference to contralateral leg			
IKDC objective, n (%)			1.000
A	23 (64%)	21 (68%)	
B	11 (31%)	9 (29%)	
C	2 (6%)	1 (3%)	

However, the rate of infrapatellar saphenous nerve lesions was significantly lower in the AL group with popliteal harvesting of the semitendinosus tendon (8.3% vs. 35.5%, *p* = 0.014).

#### Patient-reported outcome

**Fig. 5:**
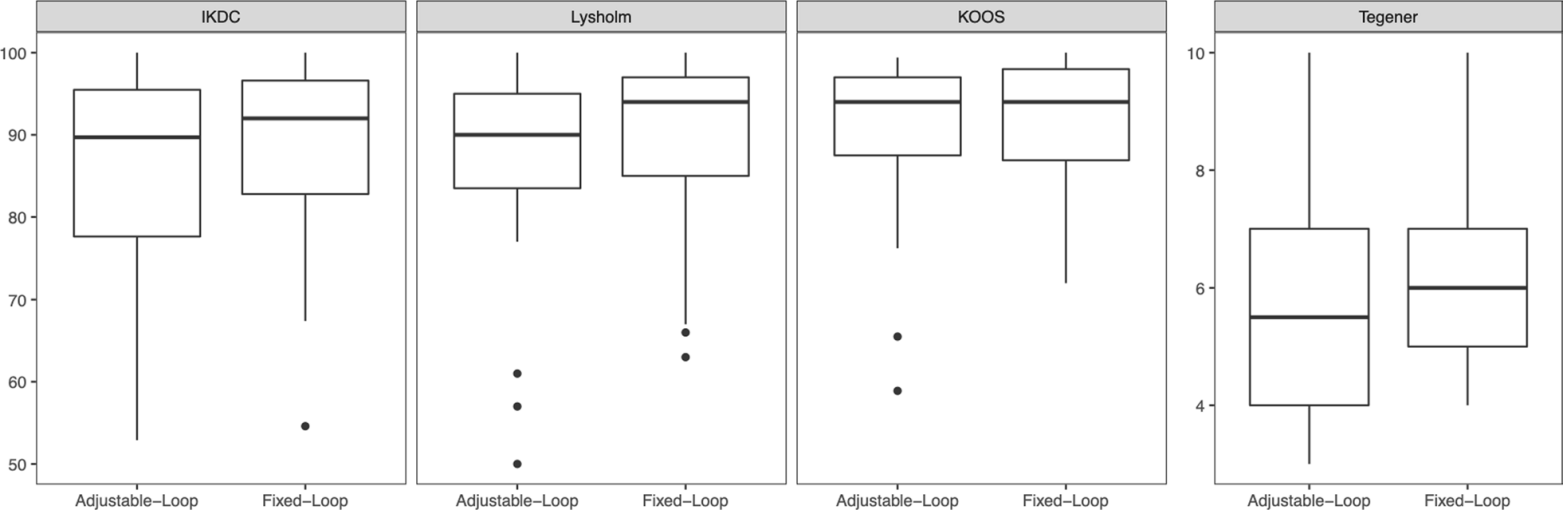
Patient-reported outcome at the time of follow-up

There were no significant differences in mean IKDC subjective score, KOOS, Lysholm score and Tegner activity scale between the groups (Fig. 5).

The mean IKDC subjective score was 88.8 ± 10.8 in the FL group and 84.8 ± 13.3 in the AL group (*p* = 0.185). The mean KOOS was 91.4 ± 8.3 in the FL group and 90.7 ± 9.3 in the AL group (*p* = 0.720). The mean Lysholm score was 89.9 ± 11.0 in the FL group and 87.3 ± 12.1 in the AL group (*p* = 0.380). The mean Tegner activity scale was 6.2 ± 1.7 in the FL group and 5.5 ± 1.7 in the AL group (*p* = 0.085).

## Discussion

The main finding of our study was that the minimally invasive all-inside technique with a femoral and tibial AL fixation resulted in similar anterior knee laxity compared to FL fixation at long-term follow-up. Furthermore, no difference was found between the AL and FL groups concerning the objective and subjective clinical outcomes. These results support the hypothesis that AL and FL suspensory fixation on the femoral and tibial side are clinically comparable.

Absolute subjective outcome scores at follow-up in our series are in a similar range to those reported in the literature (21,29–33). De Sa et al. conducted a review of AI ACL reconstruction including 526 patients with an average subjective IKDC (85.7 ± 12.2), Lysholm (92.4 ± 11.4) and Tegner score (4.9 ± 2.3) at 2 years post-op, which are comparable to our results in the AI group (21). Connaughton et al. reviewed 6 studies on AI ACL reconstruction and found a range of 83.8–89.7 for the subjective IKDC, 90.9–93.1 for the Lysholm and 5.2–6 for the Tegner score at 2 years post-op (31). Both studies conclude that AI ACL reconstruction has excellent functional and clinical outcomes similar to standard ACL reconstruction techniques.

However, numerous biomechanical studies question the stability of AL devices (14,15,34–36). In addition, Bressy et al. published insufficient results after using AL fixation on the femoral and tibial sides in a prospective trial (22). At the 12 month follow-up the mean anterior translation measured by the KT-1000 was 2.8 mm ± 2.4, and in 20% of the participants an increased translation of more than 6 mm was found. The mean subjective IKDC was 71.8% ± 16.7 and the mean Lysholm score was 79.6 ± 17.4. The authors of this study did not mention if they re-tensioned the AL system after passive cycling of the knee followed by knotting of the tensioning strands. Noonan et al. showed that these two additional steps could significantly reduce cyclic elongation using a tendon-bone implant model (13). Therefore, we would like to emphasize the importance of re-tensioning and knot tying when using AL suspensory fixation devices. This technical hint might contribute to better long-term stability and clinical outcome.

We found a significant lower rate of lesions of the infrapatellar branch of the saphenous nerve with popliteal ST tendon harvesting in the AL group as published before (24). The popliteal approach is particularly beneficial in combination with the AI technique, as only a small anteromedial skin incision is necessary for the creation of the tibial socket and placement of the tibial suture button.

The authors acknowledge that there are limitations to this study. The femoral sockets were created using two different techniques, namely anteromedial portal drilling and outside-in drilling with the same anatomical landmarks. This might lead to different femoral socket angles and graft-bending angles with a potential influence on maturation and failure of the graft (37,38). The risk of selection bias cannot be ruled out due to the retrospective design of the study as only 75 out of 305 patients were available for follow-up. As the AL technique superseded the FL technique at our institution, the follow-up time was significantly longer in the FL group. This fact and the retrospective design of the study might contribute to the higher graft failure rate in the FL group (16.2%) compared to the AL group (5.2%). However, the baseline characteristics of the two study groups were compared and we found a homogenous population regarding age, gender, and time to surgery. Furthermore, the objective clinical evaluation and the measurement of anterior knee laxity in the stress radiographs was performed by independent surgeons. Telos stress radiography was performed following a reproducible protocol at the same department. Despite the above-mentioned limitations, the current study is, to the best of our knowledge, the first comparison of adjustable- vs. fixed-loop suspensory fixation devices on the femoral *and tibial* side.

## Conclusions

In conclusion, we could demonstrate no difference in the objective and subjective outcomes of our two patient populations. In both techniques a 4-stranded ST tendon was used with suspensory fixation on the femoral and tibial side and as this study shows, similar results were achieved. Although the all-inside technique with AL fixation and popliteal ST harvesting did not demonstrate any quantifiable superiority to a technique with FL fixation and anteromedial ST harvesting, it is less invasive with a significantly lower rate of saphenous nerve lesions and might bring cosmetic benefits.

## Data Availability

The datasets used and/or analysed during the current study are available from the corresponding author on reasonable request.

## Abbreviations

ACL: Anterior cruciate ligament
AD: Anterior drawer
AI: All-inside
AL: Adjustable-loop
Dif: Differential
FL: Fixed-loop
IKDC: International knee documentation committee
KOOS: Knee injury and osteoarthritis outcome score
ROM: Range of motion
SD: Standard deviation
ST: Semitendinosus
